# Comparison of vonoprazan and proton pump inhibitors for the management of gastric post-endoscopic submucosal dissection ulcers and preventing delayed bleeding: a meta-analysis of randomized controlled trials

**DOI:** 10.3389/fmed.2026.1936609

**Published:** 2026-09-11

**Authors:** Nan Lin, Jianxiong Liu, Changqing Yang

**Affiliations:** Department of Gastroenterology, The Affiliated Hospital of Putian University, Putian, China

**Keywords:** artificial gastric ulcer, delayed bleeding, endoscopic submucosal dissection, proton pump inhibitors, vonoprazan

## Abstract

**Background:**

Delayed bleeding and artificial-ulcer healing are important clinical concerns after gastric endoscopic submucosal dissection (ESD). Vonoprazan rapidly suppresses gastric acid, but its efficacy relative to proton pump inhibitors (PPIs) remains uncertain.

**Methods:**

We searched seven databases from inception to May 31, 2026. Randomized controlled trials (RCTs) comparing vonoprazan with PPIs after gastric ESD were included. Outcomes were pooled as relative risks (RRs) or mean differences (MDs), with 95% confidence intervals (CIs).

**Results:**

Eleven RCTs reported delayed-bleeding data for 1,136 participants (580 receiving vonoprazan and 556 receiving a PPI). Delayed bleeding occurred in 16/580 (2.76%) vs. 32/556 (5.76%) participants (RR = 0.51, 95% CI 0.29–0.89; *P* = 0.02). Nine trials contributed to the relative-effect estimate because two reported zero events in both arms; a risk-difference sensitivity analysis including all 11 trials supported the result. Ulcer healing rates did not differ at 4 weeks (RR = 0.93, 95% CI 0.65–1.33; *P* = 0.69) or 8 weeks (RR = 0.98, 95% CI 0.92–1.04; *P* = 0.49). Ulcer shrinkage rates also did not differ at 4 weeks (MD = −0.89, 95% CI −2.10 to 0.33; *P* = 0.15) or 8 weeks (MD = 0.45, 95% CI −0.27 to 1.16; *P* = 0.22). Adverse events were less frequent with vonoprazan (RR = 0.57, 95% CI 0.34–0.98; *P* = 0.04).

**Conclusion:**

Vonoprazan may reduce delayed bleeding and adverse events after gastric ESD while achieving ulcer healing and shrinkage comparable to PPIs; the bleeding estimate is based on few events and should be interpreted cautiously.

**Systematic Review Registration:**

https://www.crd.york.ac.uk/PROSPERO/view/CRD420261424595, CRD420261424595.

## Introduction

With significant morbidity and mortality rates worldwide, gastric cancer continues to be a significant global health burden (1). Endoscopic submucosal dissection (ESD) is advised for certain patients with early gastric neoplasia because it allows for *en bloc* resection, precise pathological evaluation, and organ preservation (2, 3). However, ESD inevitably creates an artificial gastric ulcer. Although most post-ESD ulcers ultimately heal, the early healing phase remains clinically vulnerable. Delayed bleeding can disrupt recovery and necessitate urgent endoscopic haemostasis or transfusion (4). Reported rates vary by setting (5). In a nationwide Japanese multicentre study, bleeding after gastric ESD occurred in 4.7% and 5.0% of patients in the derivation and validation cohorts, respectively (6).

Post-ESD ulcer care is therefore integral to procedural safety after curative endoscopic resection. Acid suppression is biologically plausible because a higher intragastric pH may stabilise blood clots, reduce peptic injury to the ulcer base and support epithelial repair. This has long been the usage of proton pump inhibitors (PPIs). Evidence from endoscopically managed peptic ulcer bleeding suggests that effective acid suppression can be achieved through different administration routes in selected settings (7). However, gastric ESD creates a large iatrogenic wound rather than a conventional peptic ulcer, and the optimal pharmacological strategy remains uncertain.

Vonoprazan provides a mechanistically distinct approach to acid inhibition. In contrast to PPIs, which primarily block active proton pumps and necessitate acid activation, vonoprazan is a potassium-competitive acid blocker that inhibits stomach H+/K+ -ATPase in a reversible manner (8). Compared to traditional PPIs, it causes quick, long-lasting acid suppression and is less impacted by CYP2C19-related metabolic variability (9). These properties may be particularly relevant during the early post-ESD period, when delayed bleeding often occurs and rapid pH control may be more important than late ulcer closure.

Clinical evidence remains inconsistent. Vonoprazan wass not less effective than PPIs in avoiding delayed bleeding following gastric ESD, according to a prospective multicenter observational research by Ishida et al. Artificial ulcer healing rates were also comparable at 6 and 8 weeks (10). A large multicentre population-based study reported less post-ESD gastric bleeding with vonoprazan than with PPIs (11). Conversely, a single-centre prospective observational case-control study did not demonstrate clear superiority of vonoprazan over esomeprazole (12). In a propensity-score-matched study, Nomura et al. reported faster contraction of artificial ulcers with vonoprazan than with esomeprazole (13).

In order to evaluate vonoprazan with PPIs for the treatment of post-ESD gastric ulcers and the avoidance of delayed bleeding, we conducted a meta-analysis of randomized controlled trials (RCTs). Our goal was to offer a more solid body of evidence for the choice of acid-suppressive treatment following gastric ESD.

## Methods

### Search strategy

In compliance with the Preferred Reporting Items for Systematic Reviews and Meta-Analyses (PRISMA) 2020 statement (14), this systematic review and meta-analysis was conducted and reported. The protocol (CRD420261424595) was prospectively registered in PROSPERO. The VIP Database, Wanfang Data, PubMed, Embase, the Cochrane Library, Web of Science, and China National Knowledge Infrastructure were searched from database inception through May 31, 2026, without language restrictions. Search concepts combined vonoprazan or other acid-suppressive agents with endoscopic submucosal dissection. The complete, database-specific search syntax, fields, limits, dates, and record counts are provided in Supplementary Table S1. Because all data came from previously published studies, ethical approval was not required.

### Eligibility criteria

Research was qualified if it (1) included individuals who had early gastric cancer or precancerous gastric tumors and had undergone ESD, (2) compared postoperative vonoprazan with a PPI, and (3) were RCTs. We excluded duplicate publications, studies that did not report a prespecified outcome of interest, conference abstracts, non-RCT studies, case reports, reviews, meta-analyses, comments and letters.

### Data extraction

Two reviewers independently screened the retrieved studies according to the prespecified eligibility criteria and extracted the first author, publication year, country, study design, follow-up, outcome-specific analysis sample sizes, and treatment regimens. A third reviewer resolved disagreements through discussion. Treatment details included drug dose, administration frequency, and duration. The primary outcomes were delayed bleeding and ulcer healing at 4 or 8 weeks after ESD; secondary outcomes were ulcer shrinkage at 4 or 8 weeks and adverse events. Definitions were extracted as reported in each RCT. Delayed bleeding was generally identified by haematemesis or melaena, a clinically important decrease in haemoglobin, and/or endoscopic confirmation or haemostatic treatment. Ulcer healing was generally defined by complete closure or transition to a scar stage at the prespecified assessment. Although the operational criteria and assessment windows varied, the trials captured sufficiently similar clinical constructs to permit cautious pooling; study-level definitions are detailed in Supplementary Table S2. The ulcer shrinkage rate was calculated as [1−(ulcer size at follow-up/original ulcer size)] × 100.

### Risk-of-bias assessment

The Cochrane Collaboration's risk-of-bias tool was used by two reviewers to evaluate the risk of bias (15). Random sequence generation, allocation concealment, participant and staff blinding, outcome assessment blinding, inadequate outcome data, selective reporting, and other bias were among the evaluated areas. A low, unclear, or high risk of bias was assigned to each domain. A third reviewer was consulted or discussed in order to settle disagreements.

### Statistical analysis

RevMan version 5.4 (Nordic Cochrane Centre, Cochrane Collaboration, Copenhagen, Denmark) was used for the primary statistical analyses. Mean differences (MDs) with 95% confidence intervals (CIs) were used for continuous outcomes, and Mantel–Haenszel relative risks (RRs) with 95% CIs were used for dichotomous outcomes. Heterogeneity was assessed with the chi-squared test and quantified with I². A fixed-effect model was used when *P* > 0.10 and I^2^ ≤ 50%; otherwise, a random-effects model was used. Statistical significance was defined as a two-sided *P* < 0.05. For delayed bleeding, trials with zero events in both arms were retained in the dataset but did not contribute information to pooled relative-effect estimates. Robustness to sparse events was examined using a Mantel–Haenszel RR without a fixed continuity correction, a Mantel–Haenszel odds ratio (OR), a Peto OR, and a Mantel–Haenszel risk difference (RD) including all 11 trials. A leave-one-out analysis was also performed for the 8-week ulcer shrinkage outcome. Potential publication bias was assessed by visual inspection of funnel plots and Egger's test in Stata version 18.0 (StataCorp, College Station, TX, USA), with *P* < 0.05 indicating evidence of funnel-plot asymmetry.

## Results

### Literature search

**Figure 1:**
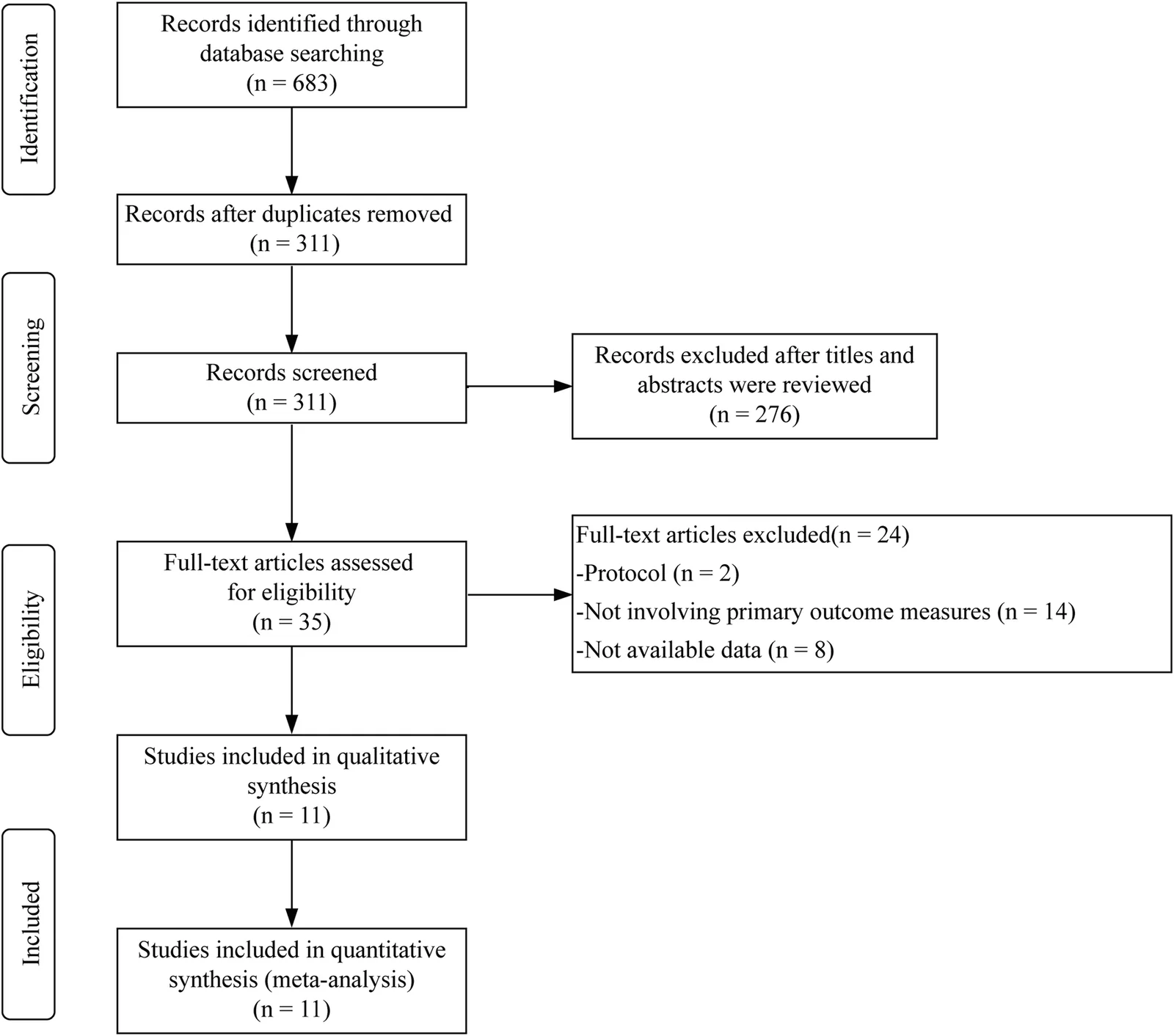
Literature search approach. RCT, randomized controlled trial.

683 records in all were found. Following the removal of 372 duplicates, 311 records were screened for titles and abstracts, and 276 were eliminated. Thirty-five reports were evaluated in their entirety. Twenty-four reports were excluded because they were protocols (*n* = 2), did not report a primary outcome (*n* = 14), or had unavailable data (*n* = 8). The meta-analysis contained eleven RCTs (16–26) (Figure 1).

### Characteristics of the included studies

Eleven RCTs (16–26), published between 2016 and 2025, were included. Ten studies (16–24, 26) were conducted in Japan and one (25) in China. All 11 trials reported delayed-bleeding data for 1,136 outcome-evaluable participants, of whom 580 received vonoprazan and 556 received a PPI. Table 1 reports the analysis denominators used for the delayed-bleeding outcome together with the study and treatment characteristics.

**Table 1:** Characteristics of the included RCTs.

Study	Publication year	Country	Study design	Follow-up (weeks)	Patients (VPZ/PPI), n	VPZ group	PPI group
Takahashi K (16)	2016	Japan	Single-centre RCT	4	26 (14/12)	VPZ 20 mg/d, 4 weeks	Lansoprazole 30 mg/d, 4 weeks
Tsuchiya I (17)	2017	Japan	Single-centre RCT	8	80 (39/41)	VPZ 20 mg/d, 8 weeks	Esomeprazole 20 mg/d, 8 weeks
Hirai A (18)	2018	Japan	Multicentre RCT	8	149 (74/75)	VPZ 20 mg/d, 8 weeks	Lansoprazole 30 mg/d, 8 weeks
Ishii Y (19)	2018	Japan	Multicentre RCT	8	53 (27/26)	VPZ 20 mg/d, Rebamipide 300 mg/d, 8 weeks	Esomeprazole 20 mg/d, Rebamipide 300 mg/d, 8 weeks
Hamada K (20)	2019	Japan	Single-centre RCT	8	139 (69/70)	VPZ 20 mg/d, 8 weeks	Lansoprazole 30 mg/d, 8 weeks
Ichida T (21)	2019	Japan	Single-centre RCT	8	82 (43/39)	VPZ 20 mg/d, Rebamipide 300 mg/d, 8 weeks	Esomeprazole 20 mg/d, Rebamipide 300 mg/d, 8 weeks
Komori H (22)	2019	Japan	Single-centre RCT	4	33 (18/15)	VPZ 20 mg/d, 4 weeks	Rabeprazole 10 mg/d, 4 weeks
Ban H (23)	2021	Japan	Single-centre RCT	8	196 (101/95)	VPZ 20 mg/d, 8 weeks	Lansoprazole 30 mg/d, 8 weeks
Kawai D (24)	2021	Japan	Single-centre RCT	8	168 (85/83)	VPZ 20 mg/d, 8 weeks	Lansoprazole 30 mg/d, 8 weeks
Gao X (25)	2024	China	Single-centre RCT	8	91 (51/40)	VPZ 20 mg/d, Rebamipide 300 mg/d, 8 weeks	Rabeprazole 40 mg/d, Rebamipide 300 mg/d, 8 weeks
Shibata H (26)	2025	Japan	Multicentre RCT	8	119 (59/60)	VPZ 20 mg/d, 8 weeks	Esomeprazole 20 mg/d, 8 weeks

PPI, proton pump inhibitor; RCT, randomized controlled trial; VPZ, vonoprazan.

### Risk of bias

**Figure 2:**
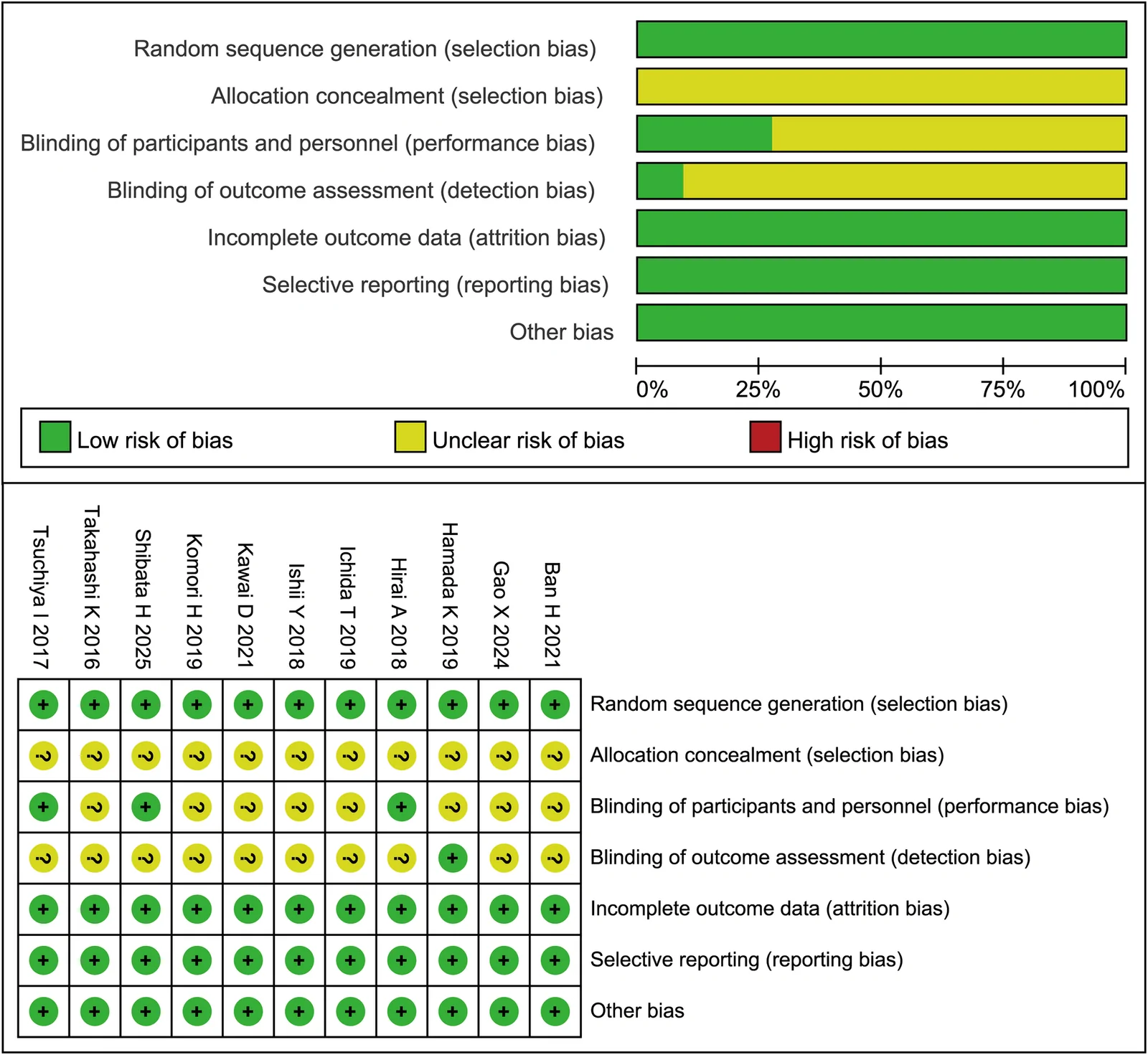
Risk-of-bias assessment for the 11 included randomized controlled trials. Colours represent the investigators’ judgements for each Cochrane risk-of-bias domain.

Random sequence generation was adequately described in all 11 studies. Allocation concealment was not sufficiently described in any study. Eight research (16, 19–25) lacked comprehensive information on participant and staff blinding, while three (17, 18, 26) did. One study (20) reported blinding of outcome assessment, whereas the remaining ten (16–19, 21–26) did not specify whether outcome assessors were blinded. There was no indication of selective reporting or other forms of bias, and all 11 studies contained complete outcome data. Figure 2 summarizes these evaluations.

### Delayed bleeding

**Figure 3:**
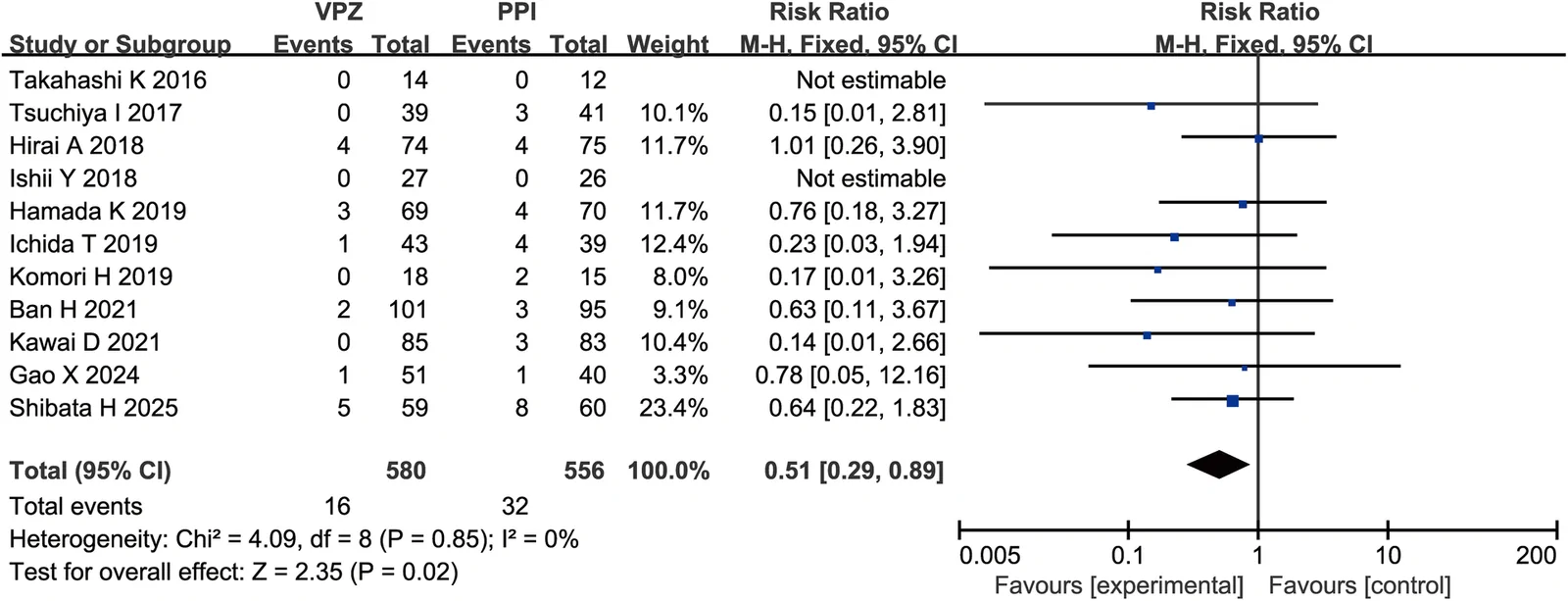
Forest plot comparing delayed bleeding after gastric ESD between vonoprazan and PPIs. Events denote participants with delayed bleeding as defined in each trial. All 11 trials reported the outcome; two double-zero trials were not estimable for the pooled RR. Boldface denotes column headers and the pooled summary row. CI, confidence interval; ESD, endoscopic submucosal dissection; PPI, proton pump inhibitor; VPZ, vonoprazan.

All 11 studies reported data on delayed bleeding following gastric ESD, comprising 580 participants in the vonoprazan group and 556 in the PPI group. Takahashi et al. and Ishii et al. reported zero events in both arms and therefore did not contribute information to the pooled RR; the remaining nine trials were informative for the relative-effect estimate. Heterogeneity was low (I^2^ = 0%, *P* = 0.85), and a fixed-effect model was used. Delayed bleeding occurred in 16/580 (2.76%) participants receiving vonoprazan and 32/556 (5.76%) receiving a PPI (RR = 0.51, 95% CI 0.29–0.89; *P* = 0.02) (Figure 3).

**Figure 4:**
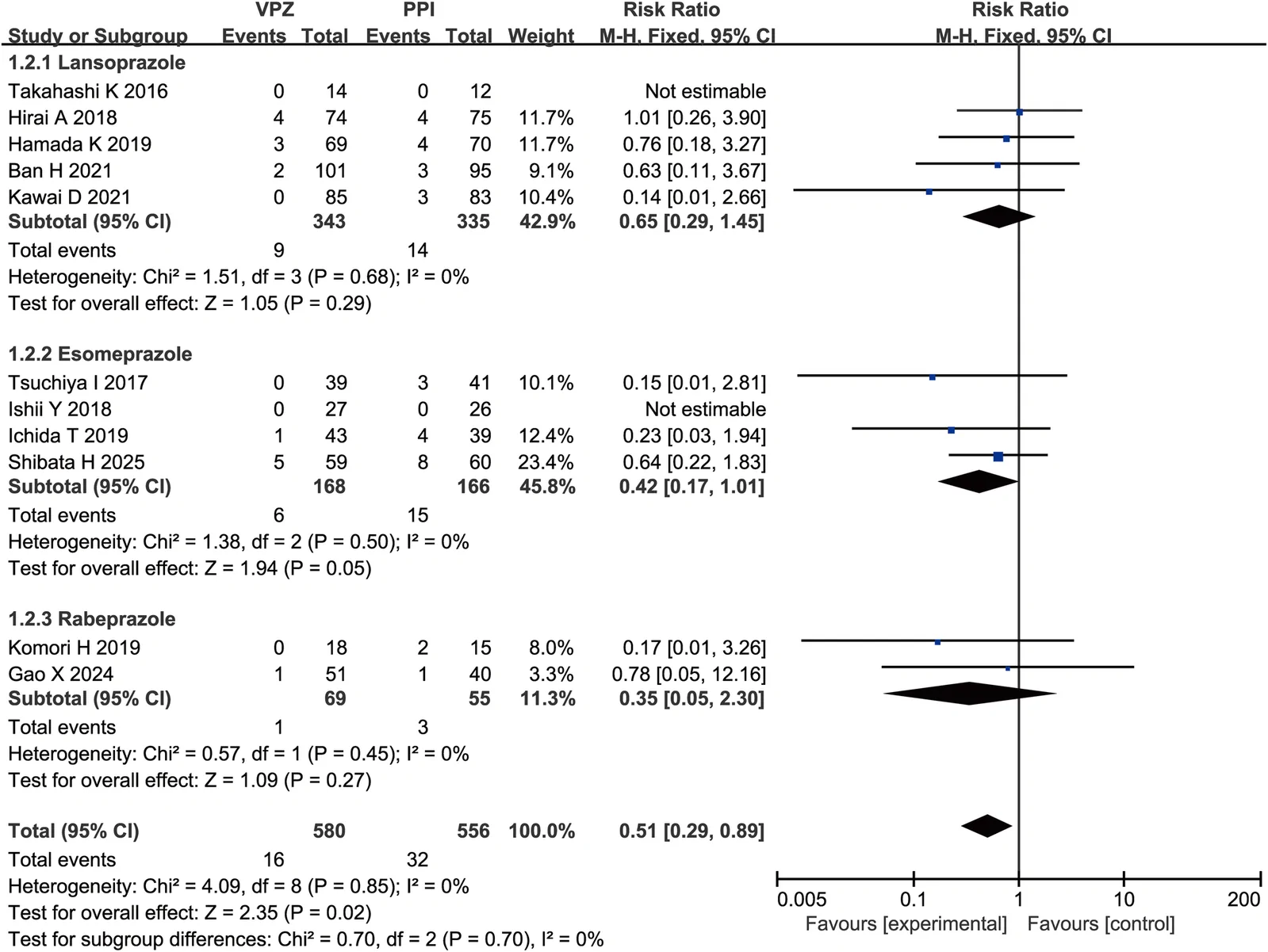
Subgroup analysis of delayed bleeding according to PPI type. Events denote participants with delayed bleeding as defined in each trial. Boldface denotes column headers, subgroup subtotal rows, and the pooled summary row. CI, confidence interval; PPI, proton pump inhibitor; VPZ, vonoprazan.

Subgroup analyses were stratified by PPI type. No statistically significant difference was observed within the lansoprazole subgroup (RR = 0.65, 95% CI 0.29–1.45; *P* = 0.29), the esomeprazole subgroup (RR = 0.42, 95% CI 0.17–1.01; *P* = 0.05), or the rabeprazole subgroup (RR = 0.35, 95% CI 0.05–2.30; *P* = 0.27). Confidence intervals were wide, and there was no evidence that the treatment effect differed by PPI type (*P* for subgroup interaction = 0.70) (Figure 4).

### Ulcer healing rate at 4 weeks after ESD

**Figure 5:**
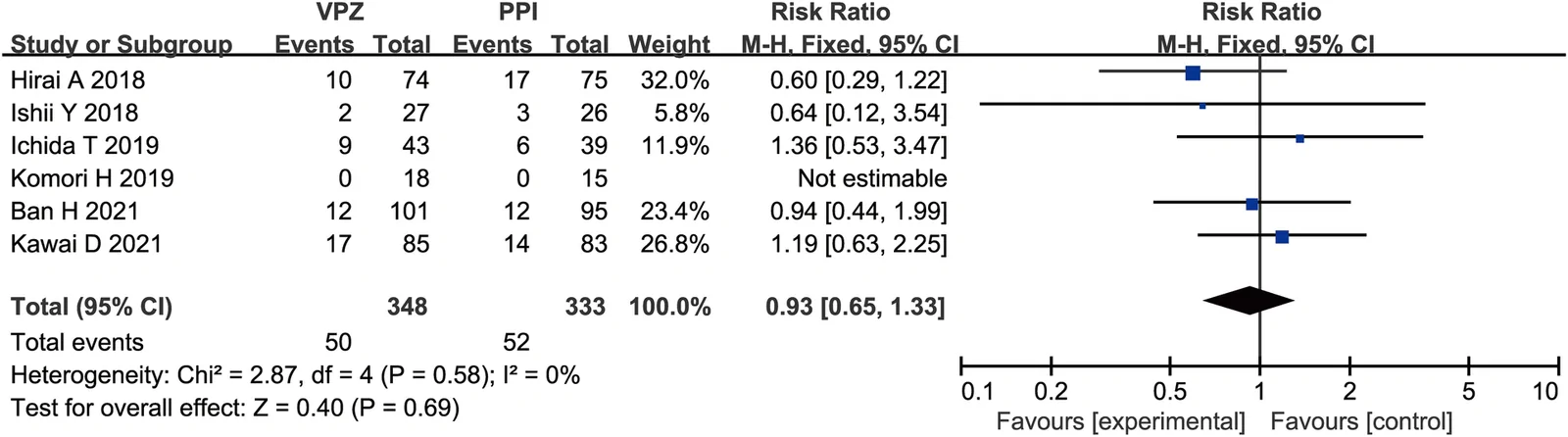
Forest plot comparing ulcer healing at 4 weeks after gastric ESD. Events denote participants whose ulcers met the healing or scar-stage criterion specified by each trial at 4 weeks. Boldface denotes column headers and the pooled summary row. CI, confidence interval; ESD, endoscopic submucosal dissection; PPI, proton pump inhibitor; VPZ, vonoprazan.

Six studies, comprising 348 patients in the vonoprazan group and 333 in the PPI group, reported the ulcer healing rate at 4 weeks. There was low heterogeneity (I^2^ = 0%, *P* = 0.58), thus a fixed-effect model was employed. No significant between-group difference was observed in 4-week ulcer healing rate (14.37% vs. 15.62%; RR = 0.93, 95% CI 0.65–1.33; *P* = 0.69) (Figure 5).

### Ulcer healing rate at 8 weeks after ESD

**Figure 6:**
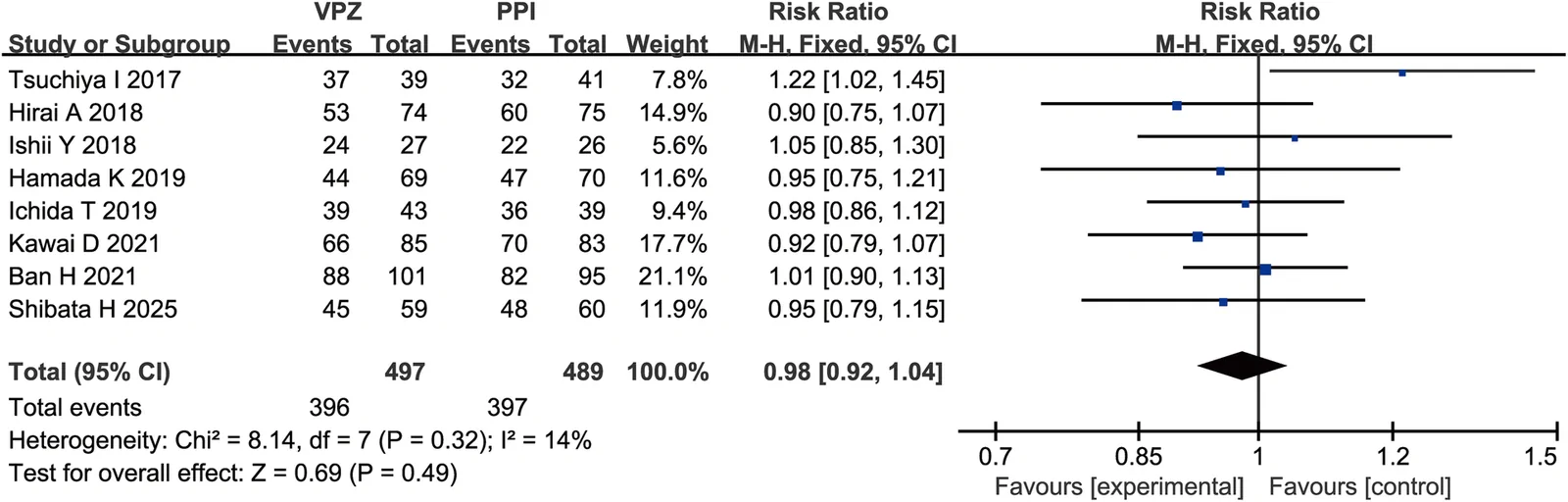
Forest plot comparing ulcer healing at 8 weeks after gastric ESD. Events denote participants whose ulcers met the healing or scar-stage criterion specified by each trial at 8 weeks. Boldface denotes column headers and the pooled summary row. CI, confidence interval; ESD, endoscopic submucosal dissection; PPI, proton pump inhibitor; VPZ, vonoprazan.

Eight trials, comprising 497 patients in the vonoprazan group and 489 in the PPI group, reported the ulcer healing rate at 8 weeks. There was low heterogeneity (I² = 14%, *P* = 0.32), thus a fixed-effect model was employed. No significant between-group difference was observed in 8-week ulcer healing rate (79.68% vs. 81.19%; RR = 0.98, 95% CI 0.92–1.04; *P* = 0.49) (Figure 6).

**Figure 7:**
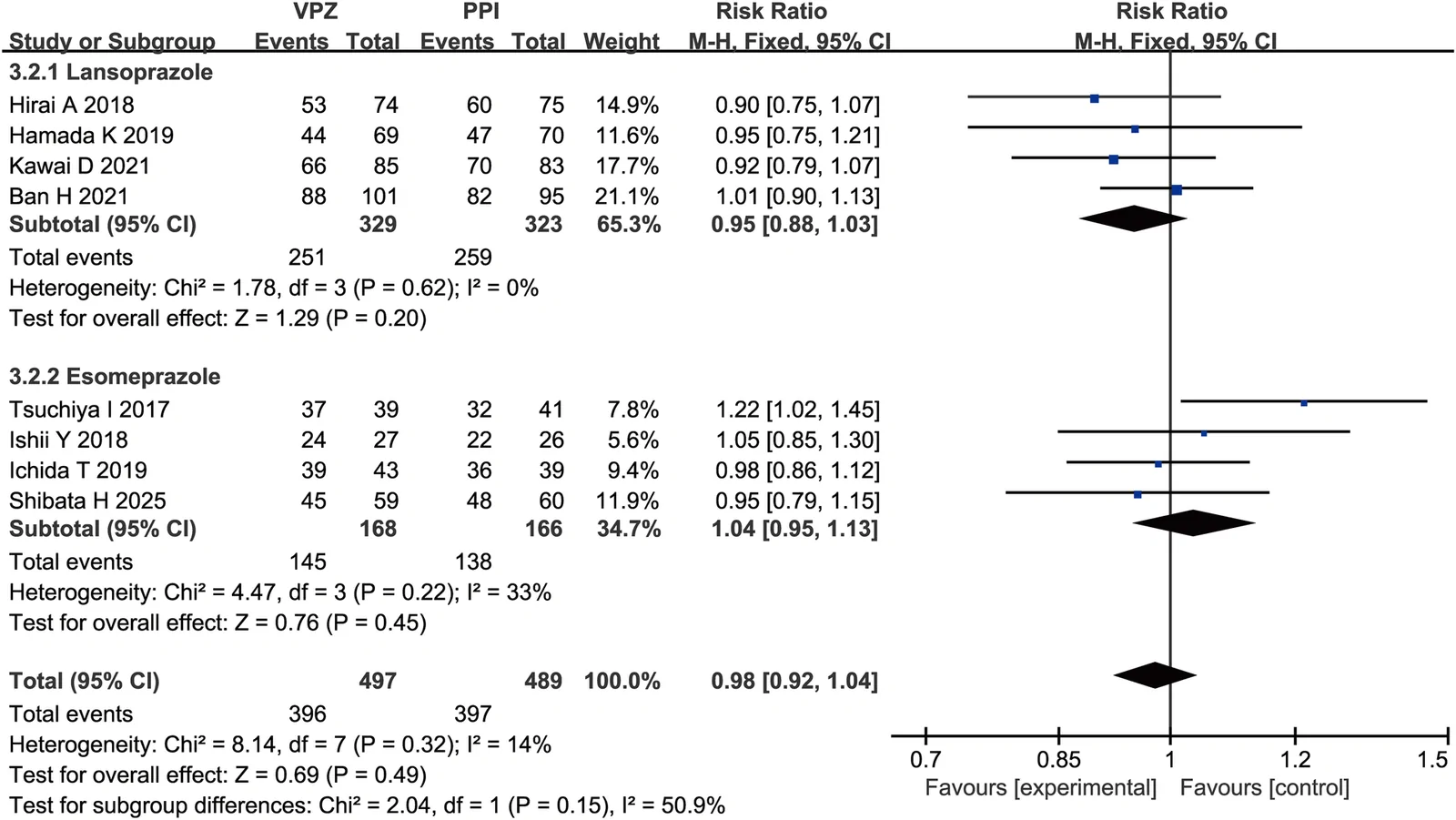
Subgroup analysis of ulcer healing at 8 weeks according to PPI type. Events denote participants whose ulcers met the healing or scar-stage criterion specified by each trial. Boldface denotes column headers, subgroup subtotal rows, and the pooled summary row. CI, confidence interval; PPI, proton pump inhibitor; VPZ, vonoprazan.

Subgroup analyses were stratified by PPI type. In the lansoprazole subgroup, heterogeneity was low (I^2^ = 0%, *P* = 0.62), with no significant difference in 8-week ulcer healing rate (76.29% vs. 80.19%; RR = 0.95, 95% CI 0.88–1.03; *P* = 0.20). In the esomeprazole subgroup, there was low heterogeneity (I^2^ = 33%, *P* = 0.22) and no significant difference between the groups (86.31% vs. 83.13%; RR = 1.04, 95% CI 0.95–1.13; *P* = 0.45) (Figure 7).

### Ulcer shrinkage rate after ESD

**Figure 8:**
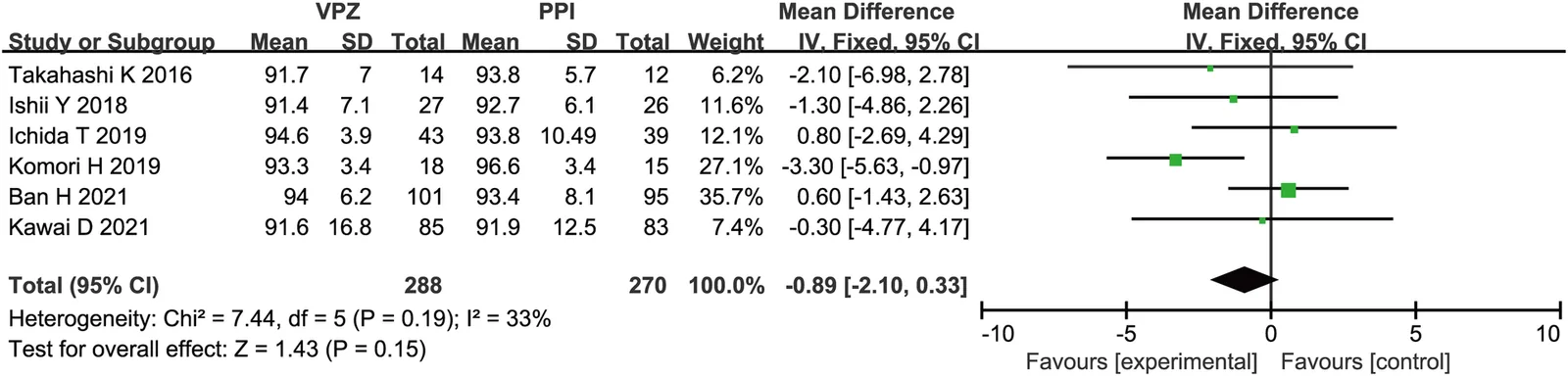
Forest plot comparing ulcer shrinkage at 4 weeks after gastric ESD. Mean differences are calculated as vonoprazan minus PPI; negative values favour vonoprazan. A fixed-effect inverse-variance model was used. Boldface denotes column headers and the pooled summary row. CI, confidence interval; ESD, endoscopic submucosal dissection; IV, inverse variance; MD, mean difference; PPI, proton pump inhibitor; VPZ, vonoprazan.

Six studies, involving 288 participants in the vonoprazan group and 270 in the PPI group, reported the 4-week ulcer shrinkage rate. Heterogeneity was modest (I^2^ = 33%, *P* = 0.19), and a fixed-effect model was used. The 4-week ulcer shrinkage rate did not differ significantly between groups (MD = −0.89, 95% CI −2.10 to 0.33; *P* = 0.15) (Figure 8).

**Figure 9:**
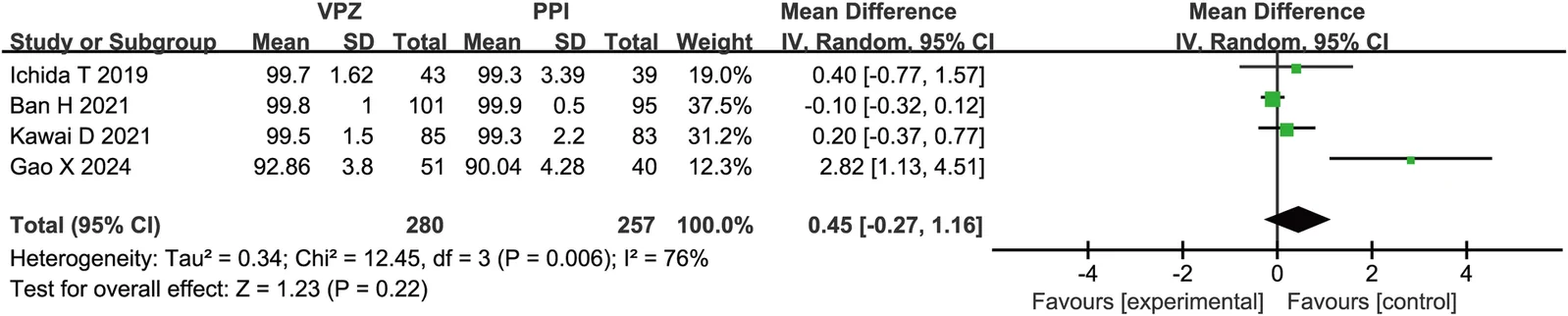
Forest plot comparing ulcer shrinkage at 8 weeks after gastric ESD. Mean differences are calculated as vonoprazan minus PPI; negative values favour vonoprazan. A random-effects inverse-variance model was used. Boldface denotes column headers and the pooled summary row. CI, confidence interval; ESD, endoscopic submucosal dissection; IV, inverse variance; MD, mean difference; PPI, proton pump inhibitor; VPZ, vonoprazan.

Four studies, involving 280 participants in the vonoprazan group and 257 in the PPI group, reported the 8-week ulcer shrinkage rate. Substantial heterogeneity was present (I^2^ = 76%, *P* = 0.006), and a random-effects model was used. The 8-week ulcer shrinkage rate did not differ significantly between groups (MD = 0.45, 95% CI −0.27 to 1.16; *P* = 0.22) (Figure 9).

### Adverse events

**Figure 10:**
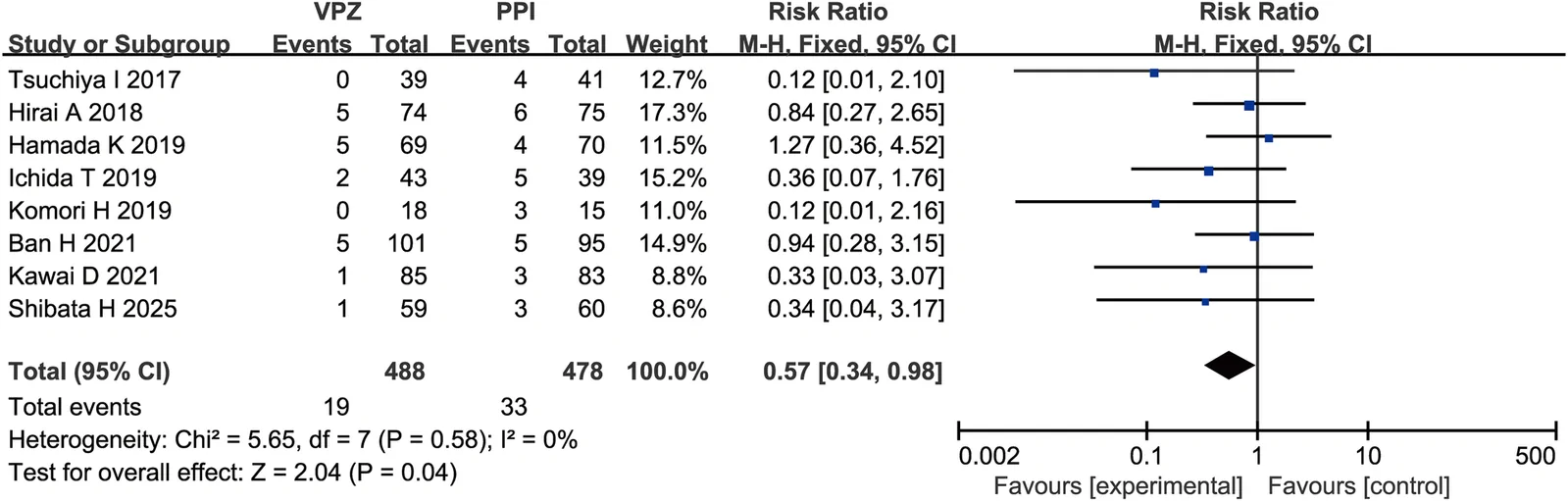
Forest plot comparing adverse events between vonoprazan and PPIs after gastric ESD. Events denote participants with at least one adverse event as reported by each trial. Boldface denotes column headers and the pooled summary row. CI, confidence interval; ESD, endoscopic submucosal dissection; PPI, proton pump inhibitor; VPZ, vonoprazan.

Adverse events were documented in eight studies, involving 488 patients in the vonoprazan group and 478 in the PPI group. There was low heterogeneity (I^2^ = 0%, *P* = 0.58), thus a fixed-effect model was employed. Vonoprazan had fewer adverse events than PPIs (3.89% vs. 6.90%; RR = 0.57, 95% CI 0.34–0.98; *P* = 0.04) (Figure 10).

### Sensitivity analysis

**Figure 11:**
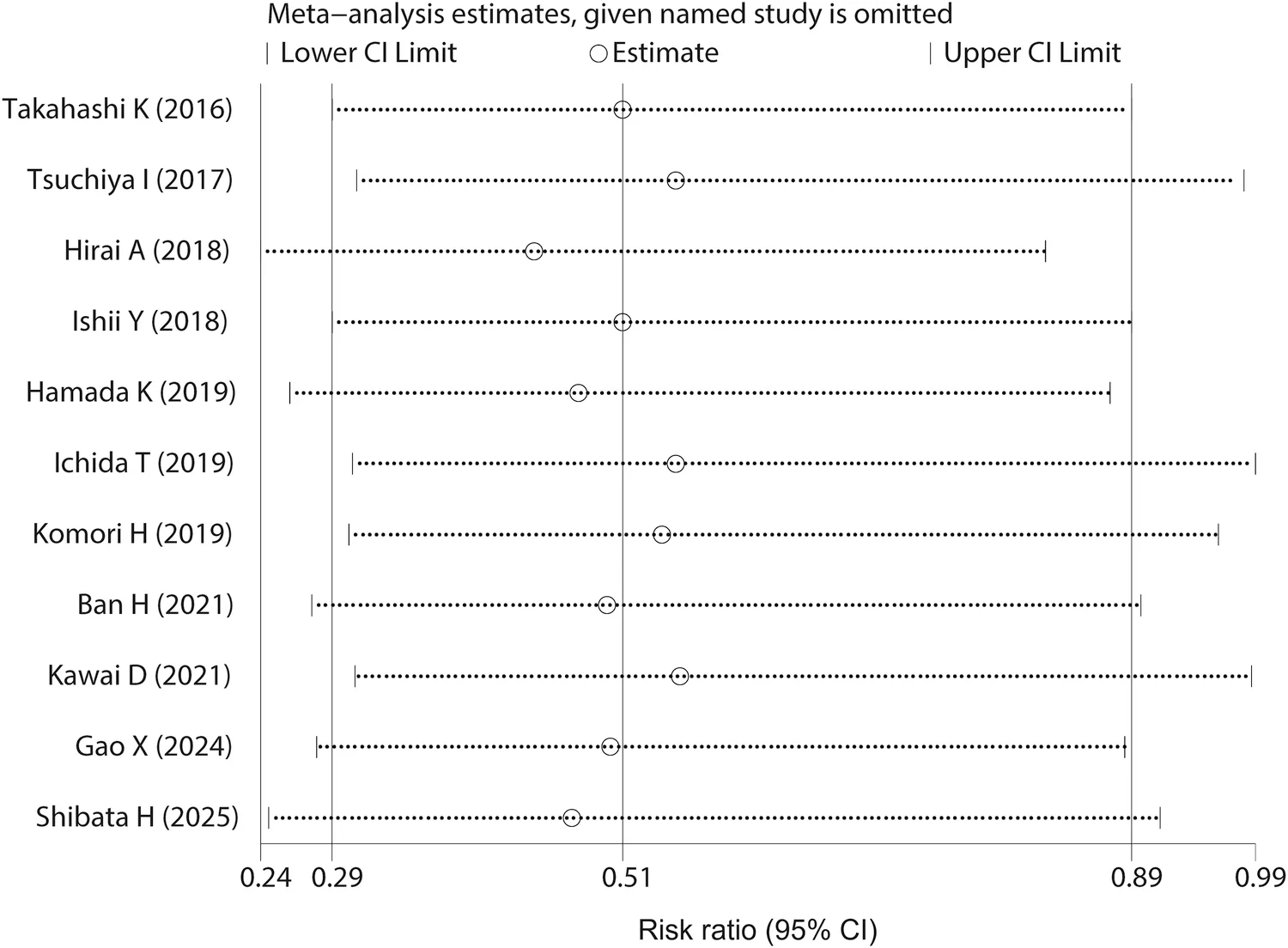
Leave-one-out sensitivity analysis for delayed bleeding. Each point represents the pooled effect after omission of the named study; the vertical reference line represents the pooled estimate from the complete analysis. CI, confidence interval.

The leave-one-out analysis of delayed bleeding did not materially alter the pooled estimate (Figure 11). Sparse-event sensitivity analyses were also consistent with the primary result: Mantel–Haenszel RR without a fixed continuity correction, 0.49 (95% CI 0.27–0.88; *P* = 0.02); Mantel–Haenszel OR, 0.47 (95% CI 0.25–0.87; *P* = 0.02); and Peto OR, 0.48 (95% CI 0.27–0.86; *P* = 0.01). The Mantel–Haenszel RD including all 11 trials was −2.92 percentage points (95% CI −5.24 to −0.60; *P* = 0.01) (Supplementary Figure S1 and Supplementary Table S3). For 8-week ulcer shrinkage, omitting Gao et al. reduced I^2^ from 76% to 0% and yielded an MD of −0.05 (95% CI −0.25 to 0.15), whereas omission of each other study left substantial heterogeneity (I^2^ = 76%–83%) (Supplementary Table S4).

### Publication bias

**Figure 12:**
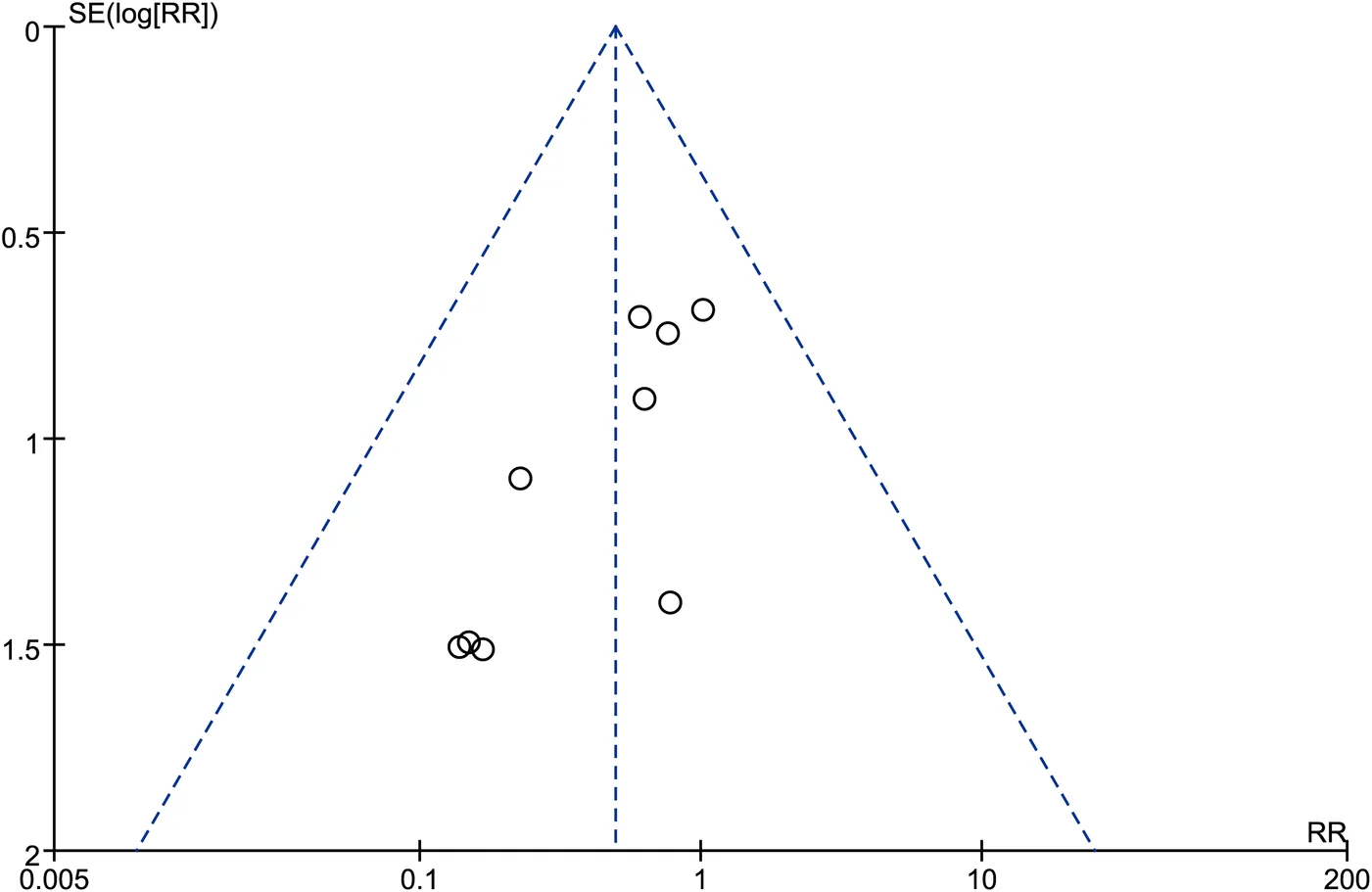
Funnel plot for the delayed-bleeding analysis. Each point represents one trial, with the treatment-effect estimate plotted against its standard error. RR, risk ratio; SE, standard error.

If the meta-analysis includes more than ten papers, publication bias can be assessed using a quantitative study using Egger's test and a qualitative inspection of the funnel plot. The analysis of delayed bleeding had a *P*-value of 0.22, without significant bias (Figure 12).

## Discussion

This meta-analysis of RCTs found that vonoprazan was associated with a lower pooled risk of delayed bleeding after gastric ESD than PPIs. Ulcer healing and shrinkage outcomes were not significantly different between treatments, while fewer adverse events were reported with vonoprazan. The delayed-bleeding result was based on 48 events (16 vs. 32); two double-zero trials did not inform the pooled RR. Nevertheless, the direction and statistical inference were consistent across alternative sparse-event methods, including an RD analysis that retained all 11 trials. Previous observational evidence similarly reported a lower risk of delayed bleeding with vonoprazan after propensity-score matching (27), and a previous meta-analysis of randomized and retrospective studies found no significant difference in ulcer healing at 4 or 8 weeks (28). Restricting the present analysis to RCTs reduces confounding, although the limited number of bleeding events remains important.

The lower pooled risk of delayed bleeding with vonoprazan may be related to its mechanism of acid inhibition. Gastric H+/K+ -ATPase is reversibly and competitively inhibited by vonoprazan, a potassium-competitive acid blocker (29). Although events can happen up to two weeks following the operation, the majority of delayed bleeding happens within 48 h of ESD (30). Rapid elevation of intragastric pH may therefore be particularly relevant during this period. PPIs typically require 3–5 days to achieve their maximal acid-inhibitory effect. Vonoprazan, on the other hand, offers potent, long-lasting acid suppression from the first dose and does not require acid-induced activation (31). This distinction may be clinically relevant because delayed bleeding often occurs before complete ulcer epithelialisation (32). Stabilisation of blood clots and protection of exposed vessels may be more important at this stage than later scar formation. Differences in drug metabolism may also contribute. The primary metabolizers of vonoprazan are CYP3A4/5 and, to a lesser degree, CYP2B6, CYP2C19, CYP2D6, and SULT2A1 (33). Hepatic CYP2C19, which primarily inactivates PPIs, exhibits significant genetic and phenotypic heterogeneity that may affect therapy response (34).

The absence of significant differences in ulcer healing and shrinkage also warrants consideration. Artificial ulcers after ESD differ from conventional peptic ulcers, and stronger acid suppression does not necessarily accelerate complete post-ESD healing once adequate pH control has been achieved (35–39). In the delayed-bleeding subgroup analysis, the pooled effect within each individual PPI subgroup was not statistically significant, but these estimates had wider CIs than the overall estimate and the interaction test was not significant (*P* = 0.70). Thus, the data do not demonstrate that the effect differs by PPI type and do not establish independent superiority over each specific PPI. For 8-week shrinkage, correction of the Komori et al. time point left four studies and substantial heterogeneity. The leave-one-out analysis identified Gao et al. as the principal driver: its omission reduced I² to 0%. Plausible sources include differences in ulcer measurement or shrinkage calculation, concomitant rebamipide, the rabeprazole dose of 40 mg/day, and treatment or assessment timing. Because these factors could not be separated with the available aggregate data, the random-effects result should be interpreted cautiously rather than using heterogeneity alone to exclude the study.

Fewer adverse events were reported with vonoprazan than with PPIs. This finding is encouraging, but adverse-event definitions and reporting were not consistent across studies. Most trials were designed primarily to evaluate ulcer healing or bleeding, rather than rare adverse events or long-term safety signals. The present findings therefore support favourable short-term tolerability but do not establish the long-term comparative safety of vonoprazan. Future RCTs should predefine adverse-event categories and systematically collect laboratory and symptom-related outcomes to clarify the long-term safety profile.

This study has several limitations. First, delayed bleeding was uncommon, and two double-zero trials could not inform relative-effect estimates; consequently, the pooled RR was derived from nine informative trials and had limited precision. Second, operational definitions and assessment windows for delayed bleeding and ulcer healing varied across trials despite addressing similar clinical constructs. Third, most trials were conducted in Japan and only one in China, limiting generalisability to other populations. Fourth, PPI type, dose, treatment duration, use of mucosal protective agents, and ulcer measurement methods differed. These differences were particularly relevant to the substantial heterogeneity in the 8-week shrinkage analysis. Finally, subgroup estimates by PPI type were imprecise, and available data were insufficient for robust analyses by antithrombotic therapy, lesion location, resection area, or Helicobacter pylori status.

## Conclusion

In summary, vonoprazan was associated with a lower pooled risk of delayed bleeding after gastric ESD and with fewer reported adverse events, while ulcer healing and shrinkage were comparable to PPIs. Because delayed-bleeding events were sparse and subgroup estimates were imprecise, the magnitude and PPI-specific consistency of the benefit require confirmation in adequately powered trials.

## Data Availability

The raw data supporting the conclusions of this article will be made available by the authors, without undue reservation.
