# Supplementary material for: Comparison of vonoprazan and proton pump inhibitors for the management of gastric post-endoscopic submucosal dissection ulcers and preventing delayed bleeding: a meta-analysis of randomized controlled trials
Source: Front Med (Lausanne). 2026 Sep 11;13:1936609. doi: 10.3389/fmed.2026.1936609 (PMC13612255; doi:10.3389/fmed.2026.1936609)
Supplement: Supplementary file 1 [file Table1.docx]

**Supplementary Tables**

Manuscript ID: 1936609 | Frontiers in Medicine | Systematic Review

# Supplementary Table S1. Complete database search strategies

| **Database/platform** | **Exact search syntax** | **Field(s)** | **Coverage start** | **Last search** | **Limits** | **Records** |
| --- | --- | --- | --- | --- | --- | --- |
| Cochrane Library | "Vonoprazan" AND "endoscopic submucosal dissection" | All fields | Inception | 31 May 2026 | No language restriction | 40 |
| PubMed | (("Endoscopic Submucosal Dissection"[Title/Abstract] OR ESD[Title/Abstract]) AND (proton pump inhibitors[Title/Abstract] OR proton pump inhibitor[Title/Abstract] OR omeprazole[Title/Abstract] OR lansoprazole[Title/Abstract] OR esomeprazole[Title/Abstract] OR rabeprazole[Title/Abstract] OR pantoprazole[Title/Abstract] OR ilaprazole[Title/Abstract] OR potassium ion competitive acid blockers[Title/Abstract] OR potassium ion competitive acid blocker[Title/Abstract] OR vonoprazan[Title/Abstract] OR tegoprazan[Title/Abstract] OR keverprazan[Title/Abstract])) | Title/Abstract | Inception | 31 May 2026 | No language restriction | 199 |
| Web of Science | TI=(vonoprazan AND "endoscopic submucosal dissection") | Title | Inception | 31 May 2026 | No language restriction | 94 |
| Embase | "Vonoprazan" AND "endoscopic submucosal dissection" | All fields | Inception | 31 May 2026 | No language restriction | 91 |
| Wanfang Data | 全部:(伏诺拉生 OR "拉唑") and 全部:(剥离) | All fields | Inception | 31 May 2026 | No language restriction | 87 |
| CNKI | （篇关摘：伏诺拉生 + "拉唑"(模糊)）AND（篇关摘：剥离(模糊)） | Title/keywords/abstract | Inception | 31 May 2026 | No language restriction | 97 |
| VIP Database | 题名或关键词=伏诺拉生 OR 拉唑 AND 题名或关键词=内镜 | Title or keywords | Inception | 31 May 2026 | No language restriction | 75 |
| Total | — | — | — | — | — | 683 |

# Supplementary Table S2. Study-level definitions of delayed bleeding and ulcer healing

| **Study** | **Delayed-bleeding definition** | **Assessment window** | **Ulcer-healing definition/time point** |
| --- | --- | --- | --- |
| Takahashi K 2016 | Delayed bleeding was monitored, but no operational definition was reported. | Through 4 weeks | Healing was evaluated at day 28 using ulcer shrinkage; delayed healing was defined as <90% shrinkage. |
| Tsuchiya I 2017 | Haematemesis or melaena and/or a haemoglobin decrease ≥2 g/dL. | Through 8 weeks | Scar stage S1 or S2 at 8 weeks. |
| Hirai A 2018 | Haematemesis or melaena requiring haemostatic treatment plus a haemoglobin decrease >2 g/dL. | Through 4 weeks | Ulcer area <3 mm² or scar-stage assessment at 4 and 8 weeks. |
| Ishii Y 2018 | Haematemesis or melaena and/or the haemoglobin criterion reported by the trial. | Window not explicitly stated | Scar stage S1 or S2 at 4 and 8 weeks. |
| Hamada K 2019 | Clinical and haemoglobin criteria with endoscopic confirmation. | Through day 28 | No mucosal defect or white coat at 8 weeks. |
| Ichida T 2019 | Bleeding requiring emergency endoscopy or transfusion with a haemoglobin decrease >2 g/dL. | Window not explicitly stated | Scar stage at 4 and 8 weeks. |
| Komori H 2019 | Haematemesis or melaena or a haemoglobin decrease >2 g/dL. | Through 4 weeks | Scar stage S1 or S2 at 4 weeks. |
| Ban H 2021 | Bleeding requiring endoscopic haemostasis. | Through 4 weeks | Scar stage, complete closure, or 100% reduction at 4 and 8 weeks. |
| Kawai D 2021 | Postoperative bleeding; no further operational definition was reported. | Window not explicitly stated | Scar stage S1 or S2 at 4 and 8 weeks. |
| Gao X 2024 | Haematemesis or melaena requiring haemostasis plus a haemoglobin decrease >2 g/dL. | Through 4 weeks | Scar-stage healing at 8 weeks. |
| Shibata H 2025 | Haematemesis or tarry stool with endoscopic confirmation. | Through 8 weeks | Scar stage at 8 weeks. |

# Supplementary Table S3. Sparse-event sensitivity analyses for delayed bleeding

| **Method** | **Estimate** | **95% CI** | **P value** | **Trial contribution** |
| --- | --- | --- | --- | --- |
| Primary Mantel–Haenszel RR | 0.51 | 0.29–0.89 | 0.019 | 9 informative for relative effect |
| Mantel–Haenszel RR without fixed continuity correction | 0.49 | 0.27–0.88 | 0.016 | 9 informative for relative effect |
| Mantel–Haenszel OR without fixed continuity correction | 0.47 | 0.25–0.87 | 0.016 | 9 informative for relative effect |
| Peto OR | 0.48 | 0.27–0.86 | 0.014 | 9 informative for relative effect |
| Mantel–Haenszel RD (percentage points) | −2.92 | −5.24 to −0.60 | 0.014 | All 11 trials |

# Supplementary Table S4. Leave-one-out sensitivity analysis for 8-week ulcer shrinkage

| **Study omitted** | **MD** | **95% CI** | **I²** |
| --- | --- | --- | --- |
| Ichida T 2019 | 0.52 | −0.36 to 1.40 | 83.5% |
| Ban H 2021 | 0.97 | −0.38 to 2.32 | 75.6% |
| Kawai D 2021 | 0.85 | −0.65 to 2.35 | 83.0% |
| Gao X 2024 | −0.05 | −0.25 to 0.15 | 0.0% |

# Supplementary Figure S1

Sparse-event sensitivity analyses for delayed bleeding. Panel A compares relative-effect estimates obtained with alternative methods. Panel B shows the Mantel–Haenszel risk difference including all 11 trials, expressed in percentage points. CI, confidence interval; MH, Mantel–Haenszel; OR, odds ratio; PPI, proton pump inhibitor; RD, risk difference; RR, risk ratio; VPZ, vonoprazan.
